# Design of two ongoing clinical trials of tolvaptan in the treatment of pediatric patients with autosomal recessive polycystic kidney disease

**DOI:** 10.1186/s12882-023-03072-x

**Published:** 2023-02-13

**Authors:** Djalila Mekahli, Max C. Liebau, Melissa A. Cadnapaphornchai, Stuart L. Goldstein, Larry A. Greenbaum, Mieczyslaw Litwin, Tomas Seeman, Franz Schaefer, Lisa M. Guay-Woodford

**Affiliations:** 1 PKD Research Group, Department of Cellular and Molecular Medicine, KU Leuven, Leuven, Belgium; 2 Department of Pediatric Nephrology, University Hospitals Leuven, Herestraat 49, 3000 Leuven, Belgium; 3 Department of Pediatrics, Center for Family Health, Center for Rare Diseases, and Center for Molecular Medicine, University Hospital Cologne and Faculty of Medicine, University of Cologne, Cologne, Germany; 4 Rocky Mountain Pediatric Kidney Center, Rocky Mountain Hospital for Children at Presbyterian/St. Luke’s Medical Center, Denver, CO USA; 5 Center for Acute Care Nephrology, Cincinnati Children’s Hospital Medical Center, University of Cincinnati College of Medicine, Cincinnati, OH USA; 6 Department of Pediatrics, Division of Pediatric Nephrology, Emory University School of Medicine and Children’s Healthcare of Atlanta, Atlanta, GA USA; 7 Department of Nephrology, Kidney Transplantation and Arterial Hypertension, Children’s Memorial Health Institute, Warsaw, Poland; 8 Department of Pediatrics, 2nd Faculty of Medicine, Charles University, Prague, Czech Republic; 9 Department of Pediatrics, University Hospital Ostrava, Ostrava, Czech Republic; 10 Division of Pediatric Nephrology, University Children’s Hospital Heidelberg, Heidelberg, Germany; 11 Center for Translational Research, Children’s National Research Institute, Washington, DC USA

**Keywords:** Autosomal recessive polycystic kidney disease (ARPKD), Clinical trial, Tolvaptan, Pediatric, Efficacy, Safety

## Abstract

**Purpose:**

Autosomal recessive polycystic kidney disease (ARPKD) is a hereditary condition characterized by massive kidney enlargement and developmental liver defects. Potential consequences during childhood include the need for kidney replacement therapy (KRT). We report the design of 2 ongoing clinical trials (Study 204, Study 307) to evaluate safety, tolerability, and efficacy of tolvaptan in children with ARPKD.

**Methods:**

Both trials are of multinational, multicenter, open-label design. Age range at enrollment is 28 days to < 12 weeks in Study 204 and 28 days to < 18 years in Study 307. Subjects in both studies must have a clinical diagnosis of ARPKD, and those in Study 204 must additionally have signs indicative of risk of rapid progression to KRT, namely, all of: nephromegaly, multiple kidney cysts or increased kidney echogenicity suggesting microcysts, and oligohydramnios or anhydramnios. Target enrollment is 20 subjects for Study 204 and ≥ 10 subjects for Study 307.

**Results:**

Follow-up is 24 months in Study 204 (with optional additional treatment up to 36 months) and 18 months in Study 307. Outcomes include safety, tolerability, change in kidney function, and percentage of subjects requiring KRT relative to historical data. Regular safety assessments monitor for possible adverse effects of treatment on parameters such as liver function, kidney function, fluid balance, electrolyte levels, and growth trajectory, with increased frequency of monitoring following tolvaptan initiation or dose escalation.

**Conclusions:**

These trials will provide data on tolvaptan safety and efficacy in a population without disease-specific treatment options.

**Trial registration:**

Study 204: EudraCT 2020–005991-36; Study 307: EudraCT 2020–005992-10.

**Supplementary Information:**

The online version contains supplementary material available at 10.1186/s12882-023-03072-x.

## What is known

- Autosomal recessive polycystic kidney disease (ARPKD) is associated with severe health consequences during childhood and disease-specific treatment is lacking
- Tolvaptan slows cyst growth and glomerular filtration rate decline in adults with autosomal dominant polycystic kidney disease

## What is new

- First interventional trials in the rare disease ARPKD
- Two clinical trials to assess tolvaptan safety, tolerability, and efficacy in children with ARPKD are under way
- Each trial will provide data on safety and the effects of tolvaptan on the need for kidney replacement therapy

## Introduction

Autosomal recessive polycystic kidney disease (ARPKD) is a rare, inherited condition that occurs in approximately 1 in 25,000 births and is caused in most cases by pathogenic variants in the *PKHD1* gene [1–3]. Characteristic features are the formation and growth of cysts in the distal tubules and collecting ducts of the kidney in utero, massive kidney enlargement, congenital hepatic fibrosis, and anomalies of the intrahepatic biliary ducts [4, 5]. Patients with the most aggressive phenotypes are at risk of death in the neonatal period from respiratory insufficiency due to pulmonary hypoplasia [1, 6–8]. Disease manifestations among children with less aggressive phenotypes include arterial hypertension, chronic lung disease, continued kidney enlargement, progression to chronic kidney failure (CKF) with dialysis in early life, worsening hepatic fibrosis, portal hypertension, recurrent cholangitis, and need for kidney, liver, or combined kidney-liver transplantation, which impose a heavy health burden and negative consequences for quality of life [4, 5, 9]. Feeding difficulties may arise from compression of the stomach by enlarged organs or from the effects of kidney function impairment on appetite and/or gastric motility. Inadequate nutrition, in conjunction with risk factors such as prematurity, low birth weight, repeated hospitalization, early onset CKF, chronic hypertension, and lung disease, increase the likelihood for impaired growth and neurocognitive deficits [5].

Overall, approximately 30% of ARPKD-affected children will reach CKF by age 10 years, and another 30% will require kidney replacement therapy (KRT) by age 20, although rates of kidney disease progression are highly variable among individuals [10–12]. Risk factors for early dialysis have been identified and include oligohydramnios/anhydramnios, greater kidney volume, perinatal presentation, corticomedullary involvement, and the need for assisted breathing or ventilation [11, 13, 14]. Currently there is no disease-specific pharmacotherapy for ARPKD.

The antidiuretic hormone vasopressin regulates cyst growth in polycystic kidney disease (PKD). Activation of the vasopressin V2 receptor triggers the accumulation of intracellular cyclic adenosine monophosphate (cAMP) in kidney epithelial cells, driving abnormal cellular proliferation (cyst formation) and fluid secretion into the cyst lumen (cyst expansion) [15–18]. Vasopressin V2 receptor antagonists were shown to slow cystic development and preserve kidney function in rodent models orthologous to human ARPKD, autosomal dominant polycystic kidney disease (ADPKD), and nephronophthisis, supporting the role of vasopressin in PKD pathogenesis [19–22]. The therapeutic benefits of this strategy in PKD were demonstrated in two phase 3 clinical trials of the V2 receptor antagonist tolvaptan in adults with rapidly progressive ADPKD, TEMPO 3:4 and REPRISE [23, 24]. Tolvaptan slowed rates of kidney volume growth and kidney function decline relative to placebo. More recently, tolvaptan exhibited antagonism of vasopressin activity in children with ADPKD aged 5–17 years and yielded data suggestive of decreased kidney volume growth and preservation of kidney function. Results also demonstrated safety, tolerability, and the feasibility of a weight-based dosing approach for pediatric patients [25].

Given that ADPKD and ARPKD share similar pathomechanisms [4, 26], and in view of the lack of disease-specific treatments available for ARPKD and the urgent need to improve outcomes, we are conducting two clinical trials of tolvaptan to evaluate safety and efficacy in children and adolescents with ARPKD.

## Materials and methods

### Trial objectives and design

Both trials are evaluating the safety, tolerability, efficacy, and pharmacodynamics of tolvaptan in pediatric subjects with ARPKD. Study 204 was registered as EudraCT 2020–005991-36 (26/05/2021) and as clinicaltrials.gov NCT04786574 (08/03/2021). Study 307 was registered as EudraCT 2020–005992-10 (26/05/2021) and as clinicaltrials.gov NCT04782258 (04/03/2021).

**Fig. 1:**
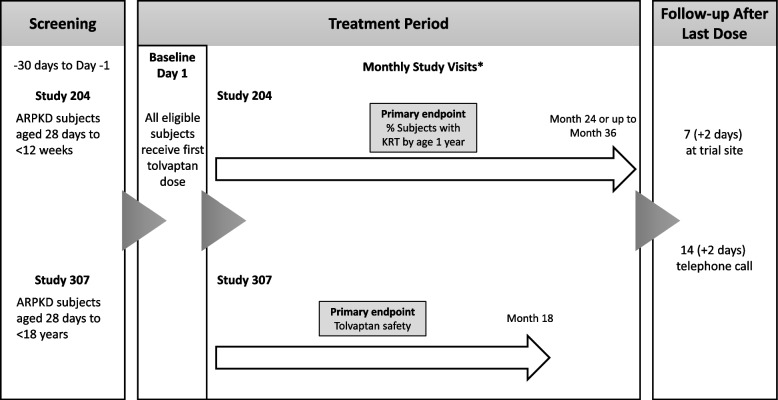
Designs of Study 204 and Study 307. *Study visits are monthly, with greater frequency of monitoring for specified outcomes (e.g., for liver enzyme levels, fluid intake, and urine volume). ARPKD, autosomal recessive polycystic kidney disease; KRT, kidney replacement therapy

Each study is a multinational, multicenter, open-label, non-randomized clinical trial. A screening period of up to 30 days to determine eligibility is followed by a tolvaptan treatment period of 24 months in Study 204 (with optional additional treatment up to 36 months) and 18 months in Study 307. A follow-up period to monitor safety continues for 14 days after the last dose of tolvaptan (Fig. 1).

In Study 204, the primary objective is to evaluate the effect of tolvaptan on the need for KRT, and the secondary objective is to evaluate changes in estimated glomerular filtration rate (eGFR) and palatability and acceptability of the tolvaptan formulation. The primary efficacy endpoint is the percentage of subjects having KRT by 1 year of age, and the main secondary efficacy endpoint is the rate of change in eGFR from pretreatment to post-treatment after 2 years of treatment.

In Study 307, the primary objective is to evaluate the safety of tolvaptan, and the secondary objective is to evaluate the effect of tolvaptan on need for KRT. The primary safety endpoint is descriptive data on adverse events, vital signs, clinical laboratory assessments, serum transaminase elevations, and change from baseline in serum sodium. Secondary endpoints are the annual rate of change in eGFR from baseline to post-treatment after 18 months of treatment; the change from baseline eGFR while on treatment at Months 1, 6, 12, and 18; time to KRT; and percentage of subjects who receive KRT. Another endpoint is change in height-adjusted total kidney volume on ultrasound to Month 18.

### Eligibility criteria

A clinical diagnosis of ARPKD is required for entry into each trial (Table 1). For Study 204, subjects are additionally required to have all the following characteristics associated with risk of KRT in the first year of life:

- Nephromegaly, and
- Detection of multiple kidney cysts or increased kidney echogenicity suggesting microcysts, and
- History of oligohydramnios or anhydramnios

**Table 1:** Eligibility criteria for subjects in Study 204 and Study 307

Both Studies	Study 204 Only	Study 307 Only
**Inclusion criteria**		
Clinical diagnosis of ARPKD		
	Age range: from 28 days to < 12 weeks	Age range: from 28 days to < 18 years
	Subjects must have all the following: • Nephromegaly, and • Detection of multiple kidney cysts or increased kidney echogenicity suggesting microcysts, and • History of oligohydramnios or anhydramnios	
Ability of parent/legal guardian to provide written, informed consent prior to initiation of any trial-related procedures, and ability, in the opinion of the principal investigator, to comply with all the requirements of the trial		Ability to provide written informed assent from all subjects old enough per local laws to provide assent
**Exclusion criteria**		
Premature birth (≤ 32 weeks gestational age) for infants 28 days to < 12 weeks of age		Females who are breast-feeding or who have a positive pregnancy test result prior to receiving tolvaptan
Anuria or KRT defined as intermittent or continuous hemodialysis, peritoneal dialysis, hemofiltration, hemodiafiltration or history of kidney transplantation		Subjects with a history of substance abuse within the last 6 months (depending on age)
Evidence of syndromic conditions associated with kidney cysts (other than ARPKD)		Subjects with a history of persistent noncompliance with antihypertensive or other important medical therapy
Abnormal liver function tests including ALT and AST, > 1.2 × ULN		Subjects who do not agree to remain abstinent or assent to use a combination of 2 of the following highly effective birth control methods for at least 28 days before the first dose of tolvaptan, during the trial (including during tolvaptan dose interruptions), and for at least 30 days after the last dose of tolvaptan: • Barrier method of contraception: condoms (male or female) with or without a spermicidal agent, diaphragm or cervical cap with spermicide • Intrauterine device • Hormone-based contraceptives which are associated with inhibition of ovulation
Has splenomegaly or portal hypertension		
Parents with kidney cystic disease		
Receiving chronic diuretic that could not be adjusted after tolvaptan initiation		
Cannot be monitored for fluid balance		
Has or at risk of having sodium and potassium electrolyte imbalances, as determined by the investigator		
Has or at risk of having significant hypovolemia (e,g., subjects that lack free access to water, without adequate fluid monitoring and management) as determined by investigator		
Clinically significant anemia, as determined by investigator		
Platelets < 50,000 µL		
Severe systolic dysfunction defined as ejection fraction < 14%		
Serum sodium levels < 130 mmol/L or > 145 mmol/L (or the ULN of the local laboratory, whichever is lower)		
Taking any other experimental medications		
Require ventilator support		
Taking medications known to induce CYP3A4		
Having an active infection including viral that would require therapy disruptive to tolvaptan dosing		
Subjects who have bladder dysfunction and/or difficulty voiding		
Subjects taking a vasopressin agonist (e.g., desmopressin)		
Subjects having concomitant illnesses or taking medications likely to confound endpoint assessments, including taking approved (i.e., marketed) therapies for the purpose of affecting PKD cysts such as tolvaptan, vasopressin antagonists, anti-sense RNA therapies, rapamycin, sirolimus, everolimus, or somatostatin analogs (i.e., octreotide, sandostatin)		
Received or are scheduled to receive a liver transplant		
History of cholangitis		
Has findings consistent with clinically significant portal hypertension (e.g., varices, variceal bleeding, hypersplenism indicated by thrombocytopenia)		

*ALT* Alanine aminotransferase, *ARPKD* Autosomal recessive polycystic kidney disease, *AST* Aspartate aminotransferase, *CYP* Cytochrome P450, *KRT* Kidney replacement therapy, *PKD* Polycystic kidney disease, *RNA* Ribonucleic acid, *SD* Standard deviation, *ULN* Upper limit of normal

Ages of eligibility are between 28 days and < 12 weeks, inclusive, for Study 204 and between 28 days and < 18 years for Study 307. The requirements for age at least 28 days and multiple characteristics indicative of risk for KRT in Study 204 were intended to enroll patients who had survived the neonatal period but were likely to need early KRT. To facilitate more rapid recruitment, genetic confirmation of *PKHD1*-associated disease is not a requirement for enrollment in either study. However, genetic testing will be performed on all enrollees, and those without *PKHD1*-confirmed disease will be discontinued from the study, with new participants recruited to reach enrollment targets.

Exclusion criteria are shown in Table 1 and include current KRT, abnormal liver function tests, presence of portal hypertension, presence or risk of electrolyte imbalances or hypovolemia, need for ventilator support, and use of medications that induce cytochrome P450 (CYP) 3A4. The criteria are essentially the same for both studies, with the exception of several criteria inapplicable to the Study 204 age group that are used only in Study 307.

### Recruitment

Given the challenges to recruitment for clinical trials in rare diseases, the trials are conducted internationally with multiple study centers. The pre-existing participation of Study 204 and Study 307 investigators in the ARegPKD and ARPKDB registry studies ensured the inclusion of important international ARPKD clinical centers in the present research [27, 28]. The distinct approaches of the ARegPKD and ARPKDB registries to patient recruitment in Europe and in the United States facilitate greater international participant outreach than a single recruitment strategy.

### Treatments

Subjects ≥ 4 years of age and who are able to swallow tablets receive oral split doses. Subjects under 4 years of age and older subjects who are unable to swallow tablets receive suspension doses. All subjects are closely monitored (e.g., fluid balance, body weight) to ensure safety and tolerability as described below. Dose escalations are timed so that liver function testing can be performed 2 weeks after a subject’s new dose.

#### Suspension

For suspension-based dosing, tolvaptan 50 mg is provided in aluminum sticks containing granules; a separate aluminum stick containing excipients to stabilize tolvaptan in the suspension is also provided. The contents of both sticks are suspended in 50 mL of water to create a 1 mg/mL suspension. Doses are administered orally or via a feeding tube if a feeding tube is in place at the time of the scheduled study drug administration.

Tolvaptan is metabolized by CYP3A4 [29]. CYP3A4 activity at birth is low relative to adult activity, but increases rapidly thereafter, with most of the increase occurring during the first year [30]. Therefore, tolvaptan dosages were designed to accommodate increased CYP3A4 activity, based on pharmacokinetic modeling to predict tolvaptan plasma concentrations for different age ranges (Table 2). Simcyp software was used to model tolvaptan plasma concentrations in healthy adults following administration of a suspension formulation. The model was then used to predict tolvaptan concentrations in pediatrics by selecting the pediatric population age ranges (with accompanying changes in physiological parameters) from the Simcyp libraries. Time above 100 ng/mL, the tolvaptan concentration at which maximal suppression of urine osmolality is observed in adults, was the target used [29]. The selected regimens for the given age ranges are predicted to produce mean tolvaptan concentrations > 100 ng/mL for about 9 h in subjects 28 days to 2 months old, 12 h in subjects > 2 months to 6 months old, and 15 h in subjects > 6 months old. Dosages can be down-titrated after consulting with the medical monitor.

**Table 2:** Tolvaptan suspension doses and dose regimens

Age Range	Total Daily Dose	Regimen
28 days to 2 months	0.15 mg/kg	QD in the AM
> 2 months to 4 months	0.30 mg/kg	QD in the AM
> 4 months to 6 months	0.5 mg/kg	QD in the AM
> 6 months to 9 months	0.75 mg/kg	Split dose, 0.5 mg/kg AM and 0.25 mg/kg 8 h later
> 9 months	1 mg/kg	Split dose, 0.67 mg/kg AM and 0.33 mg/kg 8 h later

*AM* Morning, *QD* Once daily

#### Tablets

Subjects taking tolvaptan tablets receive daily split doses (upon awakening and 8 h later) based on body weight and tolerability as reported in a pediatric ADPKD clinical trial (Online Resource 1) [25]. As with adults [31], split-dose regimens with the larger dose taken in the morning are used to minimize overnight aquaretic effects while maintaining vasopressin V2 receptor inhibition. Each tolvaptan dose is to be administered with 240 mL of water as part of the effort to maintain proper hydration status. Subjects may down-titrate at any time during the trial if the current dose is not tolerable; however, subjects are asked to stay on the highest tolerable dose possible.

#### Concomitant medications

For subjects taking suspension and needing to take a moderate CYP3A4 inhibitor (e.g., fluconazole, erythromycin), both morning and afternoon doses are to be reduced by half, with timing of doses unchanged. For subjects taking the suspension and a strong CYP3A4 inhibitor, both morning and afternoon doses (as applicable), should be reduced by dividing the dose by 4.

For subjects taking tablets and requiring moderate CYP3A4 inhibitors, doses should be divided by 2 when possible. For 15/7.5 mg and 7.5/7.5 mg regimens, only the morning dose should be taken. For 7.5 mg once daily, tolvaptan dosing should be interrupted. For subjects taking tablets and requiring strong CYP3A4 inhibitors, if total daily tolvaptan dose is ≥ 45 mg, 15 mg once daily should be taken; if not well tolerated, then tolvaptan should be down titrated to 7.5 mg once daily. If total daily dose is ≥ 22.5 mg to < 45 mg, 7.5 mg once daily should be taken, with dose interruption if not well tolerated. Treatment should be interrupted if total daily dose is < 22.5 mg.

Relevant safety assessments are performed in accordance with the institution’s local standard of care after any dosing changes for concomitant medications, including but not limited to diuretics, angiotensin converting enzyme inhibitors, and angiotensin receptor blockers.

#### Discontinuation or interruption

If subjects reach the endpoint of KRT, they discontinue treatment, and end-of-treatment visit procedures are performed. After treatment assignment, a subject may stop treatment permanently for a variety of reasons. Treatment discontinuations may be initiated by a subject’s parent/legal guardian who is not satisfied with treatment or may become medically necessary due to adverse events (e.g., liver test abnormalities meeting criteria for permanent discontinuation, see below), required treatment with a prohibited medication or therapy (Table 3), or other issues, as determined by the investigator.

**Table 3:** List of medications or therapies prohibited during the trial

1. Receiving chronic diuretic that could not be adjusted after tolvaptan initiation
2. Cytochrome P450 3A4 inducers
3. Medications or surgical therapies used for the purpose or potential for modifying the progression of polycystic kidney disease cyst growth or development. These include, but are not restricted to, somatostatin analogs (i.e., octreotide, sandostatin), anti-sense (RNA therapies, rapamycin, sirolimus, everolimus), and other vasopressin antagonists (e.g., mozavaptan, conivaptan) or agonists (e.g., desmopressin)
4. Urea, demeclocycline, lithium, or conivaptan

*RNA* Ribonucleic acid

Liver transaminase or bilirubin levels ≥ 2 × upper limit of normal (ULN) that have an uncertain or rapidly increasing trajectory should prompt at least temporary tolvaptan interruption. Tolvaptan should not be resumed until monitoring indicates abnormalities have resolved, are stable or are not rapidly increasing, and then only with an increased frequency of monitoring. In alignment with regulatory guidance [32], a subject must be discontinued from the trial on confirmation of any of the following criteria:

- Alanine aminotransferase (ALT) or aspartate aminotransferase (AST) ≥ 8 × ULN
- ALT or AST ≥ 5 × ULN for more than 2 weeks
- ALT or AST ≥ 3 × ULN and (total bilirubin ≥ 2 × ULN or International Normalized Ratio > 1.5)
- ALT or AST ≥ 3 × ULN with appearance of fatigue, nausea, vomiting, right upper quadrant pain or tenderness, fever, rash, and/or eosinophilia (> 5%) and signs of jaundice

If clinically significant adverse events are observed, tolvaptan should be interrupted, appropriate medical intervention and corrective actions should be provided to the subject, and the medical monitor should be notified. Once the underlying condition has been corrected, tolvaptan may be restarted, and a dose reduction considered after consultation with the medical monitor. If a subject is unable to have liver function testing and/or serum sodium monitoring, tolvaptan will be interrupted, and the medical monitor should be contacted. Tolvaptan can be restarted after consultation with the medical monitor and to verify that the subject is able to take fluids.

### Trial assessments

Study visits and laboratory testing are conducted monthly throughout each trial. Visits occur either in-hospital, at the trial site, or if the subject is at home via visiting nurse if sampling for liver function testing is required. If no laboratory assessments are required, other procedures can be done via telemedicine visit. Home visiting nurse services and/or telemedicine visits are used if allowed per country.

At screening, a genetic test sample is collected to verify the diagnosis and potentially conduct additional analyses related to disease progression, prognosis, treatment response, and adverse events. Genetic diagnosis is not required for trial eligibility, but the investigator will use genetic testing results to determine if the participant will continue in the trial.

Efficacy assessments are events of KRT (defined as intermittent or continuous hemodialysis, peritoneal dialysis, hemofiltration, hemodiafiltration or kidney transplantation) and kidney function using eGFR. Urine volume and fluid intake over a 24-h period are recorded during screening, baseline, once daily for the first month, and then 2 days before and after dose escalation. For subjects who are in the hospital, 24-h fluid intake and urine output are recorded daily during the first month to assess fluid balance. After the first month, fluid intake and urine output recorded 2 days before and after a tolvaptan dose escalation provide investigators necessary information to adjust a subject’s fluid intake whether the subject is in hospital or at home. If a subject is at home, instructions are provided with specific guidance on how to obtain fluid intake and urine output and weigh diapers for urinary output. There are also instructions for toilet-trained subjects or the parents/legal guardians of urinary continent subjects on when and how to record data for their 24-h fluid intake and urine output. Kidney ultrasound is conducted at screening, baseline, and monthly through Month 18 and again at Month 24/end of treatment in Study 204 and at screening, baseline, and monthly through Month 12 and again at Month 18/end of treatment in Study 307.

Safety assessments include adverse events (as reported by the parent or legal guardian), including aquaretic adverse events; change from baseline in serum creatinine; clinical laboratory tests including liver enzyme levels and electrolytes; physical examination; vital signs; concomitant medication use; and change from baseline in growth percentile trajectories for body height/length, weight, and head circumference (for subjects ≤ 2 years of age). Blood and urine samples are obtained monthly, and sparse pharmacokinetic blood samples are taken at Months 3, 6, 9, and 12. In case of limited blood availability, serum sodium, serum creatinine, and liver enzymes are prioritized. Liver enzyme testing should be performed in all subjects for close monitoring of liver function 2 weeks after the first tolvaptan dose, after each dose escalation, and monthly. Dose escalations should be timed so that liver function tests can be performed 2 weeks after a subject’s new dose. Palatability and acceptability of the suspension formulation will be collected once throughout the treatment period in subjects able to take the suspension orally.

Standardized family-centered questionnaires assessing health-related quality of life are administered during visits in both studies. Participant-centered questionnaires are administered in Study 307 as appropriate for participants.

### Statistical analyses

Study endpoints will be assessed using descriptive statistics. All safety/efficacy data will be summarized for observed (non-missing) values only. The safety and efficacy datasets for this trial are based on the intent to treat population, which consists of all subjects who were administered at least one dose of tolvaptan.

For Study 204, the primary efficacy endpoint (percentage of subjects having KRT by 1 year of age) will be compared with historical data [13] using Fisher’s exact test.

The target sample size for Study 204 is 20 subjects. In the study by Burgmaier et al., the incidence rate of dialysis for subjects with oligohydramnios/anhydramnios and enlarged kidneys and renal cysts within 12 months after birth was model-predicted as 22.3/74.4 (= 30%) [13]. The same paper also reported 107 subjects with oligohydramnios/anhydramnios, 70 subjects with enlarged kidneys, and 82 subjects with renal cysts in the ARegPKD registry trial. Incorporating all this information, an assumption may be drawn that there were 70 subjects with oligohydramnios/anhydramnios and enlarged kidneys and renal cysts in the ARegPKD registry trial. With a sample size of 20 and assuming that 5% of subjects in the current study will have dialysis within 12 months after their birth, the trial has 74% power in the historical comparison for a two-sided alpha of 0.05.

For Study 307, since the data collected will be summarized using descriptive statistics and not aimed at testing a specific hypothesis, no formal power calculations are undertaken. The target enrollment is ≥ 10 subjects.

### Ethical conduct

The trial is being performed in compliance with International Council for Harmonization Good Clinical Practice guidance, international ethical principles derived from the Declaration of Helsinki and Council for International Organizations of Medical Science guidelines, and applicable local laws and regulations. Each trial site must obtain approval/favorable opinion by an institutional review board or independent ethics committee according to regional requirements.

Informed consent is obtained from all subjects’ parents or their legal guardians. In Study 307, written informed assent is obtained from all subjects old enough per local laws to provide assent. Age-appropriate assent documents were created and subjects who are able are required to reconsent or assent as appropriate if they matriculate from one age group to another.

## Discussion

These clinical trials are expected to provide data on the safety, tolerability, and efficacy of tolvaptan treatment and determinants of progression in ARPKD. The genetic data collected may improve the ability to diagnose and predict the course of ARPKD, which are challenging given the genetic complexity of the disease [5, 12, 33].

Study 204 was conceived first, as a trial to assess tolvaptan in neonatal survivors who are at high risk of early KRT and who would have the most benefit from therapeutic intervention. Accordingly, eligibility criteria for Study 204 require an age of at least 4 weeks and the presence of 3 risk factors for rapid disease progression. The European Medicines Agency requested a second trial including older children to evaluate tolvaptan in a broader spectrum of ages. Study 307 will enroll a population up to and including age 17 years and without a requirement for characteristics associated with rapid progression, resulting in a study population that is, overall, older and has less severe phenotypes.

A historical control design was feasible based on ARegPKD Registry data by Burgmaier et al. [13, 14], and preferable to a placebo-controlled design in which some participants would not receive investigational treatment. Additionally, enrolling patients with a rare disease is challenging and unlikely to yield study sample sizes adequate for statistical comparison of active treatment and controls when the chance of requiring KRT is estimated to be approximately 30% in the affected population [13]. Limitations of the control data include the potential for selection bias of the registry, with the possibility of underreporting of the most severely affected patients receiving palliative care or dialysis as well as the most mildly affected patients, who might not be treated at tertiary care centers participating in the registry. Further, detailed clinical and genetic information were often missing from the control group [13]. The continued accumulation of registry data may enable comparison of the Study 204 and Study 307 cohorts with more precisely matched controls in the future.

Regular safety monitoring is conducted to detect potential adverse effects of treatment on a range of parameters, including liver function, kidney function, fluid balance, electrolyte levels, and growth trajectory, with more frequent assessments performed following dose initiation or escalation, changes in concomitant medications, or elevated liver enzymes to ensure prompt detection and correction of adverse events. In addition to the use of questionnaires to evaluate health-related quality of life, it is expected that some of the safety data collected (for example, regarding infections, hospitalization, and use of concomitant medications) will enable better characterization of quality of life in the studied population.

An important purpose of the trials is to evaluate liver function, as tolvaptan has been associated with risk of transaminase elevations in ADPKD [34, 35]. A defining early characteristic of ARPKD, distinct from ADPKD, is defective remodeling of the ductal plate, with consequent biliary duct ectasia and hepatic fibrosis [4, 33]. It is unknown how tolvaptan might affect liver function in the ARPKD population. Procedures for liver safety monitoring, treatment interruption or discontinuation, and potential restart of therapy were developed to minimize risk of serious liver injury.

## Supplementary Information

**Additional file 1:** **Supplementary Table 1. **Up-titration and down-titration steps for tolvaptan tablet-based dosing. 

## Data Availability

To submit inquiries related to Otsuka clinical research, or to request access to individual participant data (IPD) associated with any Otsuka clinical trial, please visit https://clinical-trials.otsuka.com/. For all approved IPD access requests, Otsuka will share anonymized IPD on a remotely accessible data sharing platform.

## Abbreviations

ADPKD: Autosomal dominant polycystic kidney disease
ALT: Alanine aminotransferase
ARPKD: Autosomal recessive polycystic kidney disease
AST: Aspartate aminotransferase
cAMP: Cyclic adenosine monophosphate
CKF: Chronic kidney failure
CYP: Cytochrome P450
eGFR: Estimated glomerular filtration rate
KRT: Kidney replacement therapy
PKD: Polycystic kidney disease
ULN: Upper limit of normal
