# Supplementary material for: Design of two ongoing clinical trials of tolvaptan in the treatment of pediatric patients with autosomal recessive polycystic kidney disease
Source: BMC Nephrol. 2023 Feb 13;24:33. doi: 10.1186/s12882-023-03072-x (PMC9926647; doi:10.1186/s12882-023-03072-x)
Supplement: Supplementary file 1 — Additional file 1: Supplementary Table 1. Up-titration and down-titration steps for tolvaptan tablet-based dosing. [file 12882_2023_3072_MOESM1_ESM.docx]

**Design of Two Ongoing Clinical Trials of Tolvaptan in the Treatment of Pediatric Patients with Autosomal Recessive Polycystic Kidney Disease**

**SUPPLEMENTARY INFORMATION**

***BMC Nephrology***

Djalila Mekahli, MD, PhD*; Max C. Liebau, MD*; Melissa A. Cadnapaphornchai, MD; Stuart L. Goldstein, MD; Larry A. Greenbaum, MD; Mieczyslaw Litwin, MD, PhD; Tomas Seeman, MD, PhD; Franz Schaefer, MD; Lisa M. Guay-Woodford, MD

*Co-first authors.

**Corresponding author**

Djalila Mekahli

Department of Pediatric Nephrology, University Hospitals Leuven

Herestraat 49, 3000 Leuven, Belgium

Email: djalila.mekahli@uzleuven.be

**Supplementary Table 1.** Up-titration and down-titration steps for tolvaptan tablet-based dosing*

| **Up-titration Steps** | | | |
| --- | --- | --- | --- |
| **Body Weight** | **Starting Dose** | | **Up-titrated Maximum Dose** |
| ≥20 kg to <45 kg | 15/7.5 mg | | 30/15 mg |
| ≥45 kg to ≤75 kg | 30/15 mg | | 45/15 mg |
| >75 kg | 45/15 mg | | 60/30 mg |
| **Down-titration Steps** | | | |
| **Current Dose** | | **Down-titration Steps** | |
| 7.5 mg once daily upon awakening | | Subject discontinues treatment | |
| 7.5/7.5 mg | | 7.5 mg once daily upon awakening | |
| 15/7.5 mg | | 7.5/7.5 mg | |
| 22.5/15 mg | | 15/7.5 mg | |
| 30/15 mg | | 22.5/15 mg | |
| 45/15 mg | | 30/15 mg | |
| 60/30 mg | | 45/15 mg | |

*Tolvaptan or placebo.

Children weighing <45 kg and 45–75 kg had up-titrated doses that were 37.5% and 50%, respectively, of the highest recommended total adult target dose (90/30 mg/day). The up-titrated dose in children and adolescents weighing >75 kg was 75% of the highest recommended adult target dose. Adults are up-titrated in 3 steps.

Table reproduced with permission from: Mekahli D et al (2023) Tolvaptan for children and adolescents with autosomal dominant polycystic kidney disease: randomized controlled trial. Clin J Am Soc Nephrol 18:36-46.
